# Dual Primary Cancer Patients With Lung Cancer as a Second Primary Malignancy: A Population-Based Study

**DOI:** 10.3389/fonc.2020.515606

**Published:** 2020-10-26

**Authors:** Congkuan Song, Donghu Yu, Yujin Wang, Qingwen Wang, Zixin Guo, Jingyu Huang, Sheng Li, Weidong Hu

**Affiliations:** ^1^ Department of Thoracic Surgery, Zhongnan Hospital of Wuhan University, Wuhan, China; ^2^ Hubei Key Laboratory of Tumor Biological Behaviors and Hubei Cancer Clinical Study Center, Wuhan, China; ^3^ Department of Biological Repositories, Zhongnan Hospital of Wuhan University, Wuhan, China; ^4^ Human Genetics Resource Preservation Center of Hubei Province, Wuhan, China

**Keywords:** lung cancer as a second primary malignancy, multiple primary cancers, lung cancer, nomogram, Surveillance, Epidemiology and End Results

## Abstract

**Background:**

Research on patients with lung cancer as a second primary malignancy (LCSPM) remains limited. This study aims to determine the clinical characteristics, prognosis, and temporal relationship of other cancers to lung cancer in these patients.

**Methods:**

This study retrospectively analyzed 3465 patients with dual primary cancers from the 5253 patients with LCSPM retrieved from the Surveillance, Epidemiology and End Results (SEER) database from 2010 to 2015.

**Results:**

2285 eligible patients were further analyzed in this study cohort with 59.3% of 1-year OS, 34.7% of 3-year OS, and 25.2% of 5-year OS. The most common first primary cancer (FPC) in dual primary cancer patients with LCSPM was prostate cancer, followed by female breast cancer and urinary bladder cancer. In the entire study population, the median interval between the two primary malignancies was 21 months (range: 3.5–52 months). Age, sex, FPC location, surgery, stage, and histology of lung cancer were regarded as independent prognostic factors for these patients. The prognosis of patients with urinary bladder cancer as FPC was the worst in the univariate (*p* = 0.024) and multivariate (*p* < 0.001) Cox analyses. Lung cancer-directed surgery could significantly improve long-term survival (HR = 0.22, *p* < 0.001). Additionally, the C-index of the established nomogram with acceptable calibration curves was 0.760 (95% CI: 0.744–0.776) in the training cohort and was 0.759 (95% CI: 0.737–0.781) in the validation cohort, showing an ideal model discrimination ability. The corresponding decision curve analysis (DCA) indicated the nomogram had relatively ideal clinical utility.

**Conclusions:**

Cancer patients still have the risk of developing a new primary lung cancer. Close, lifelong follow-up is recommended for all these patients. Early detection for surgical treatment will significantly improve the prognosis of dual primary cancer patients with LCSPM. The nomogram developed to predict 1-, 3-, and 5-year OS rates has relatively good performance.

## Introduction

Lung cancer poses a serious threat to public health due to its high morbidity and mortality. Nevertheless, little attention has been paid to multiple primary cancers (MPC) involving lung cancer. With the advancement of medical technology and the extension of survival time of cancer patients, more and more cancer patients develop one or more new primary malignant tumors in the same or other organs during follow-up. MPC involving lung cancer is common clinically. Depending on incomplete statistics, the incidence of MPC involving lung cancer ranges from 0.9% to 26.3% (1–4). However, research on MPC involving lung cancer is still limited. People still do not have a clear idea of these patients. When patients have multiple primary malignancies at the same time, it is complicated for clinicians to judge the prognosis of these patients. Although the TNM staging system is the most widely used method for evaluating prognosis, it still has some limitations, especially for patients with multiple primary malignancies (they tend to have special biological characteristics different from single primary malignancy). Thus, it is necessary to learn more about this particular disease and seek more refined methods to predict the survival of these patients. Nomogram, which has been widely used to evaluate the prognosis of cancer patients in recent years owing to its convenience and accuracy (5, 6), may be a good choice for this purpose. This study is to conduct a retrospective analysis based on the clinical information of LCSPM patients to understand the common site distribution of the first primary cancer (FPC) and time interval between two primary malignancies and to determine the prognostic factors and to develop a nomogram that can predict the survival in order to provide certain evidence for guiding clinical practice.

## Materials and Methods

### Data Source and Variable Selection

**Figure 1:**
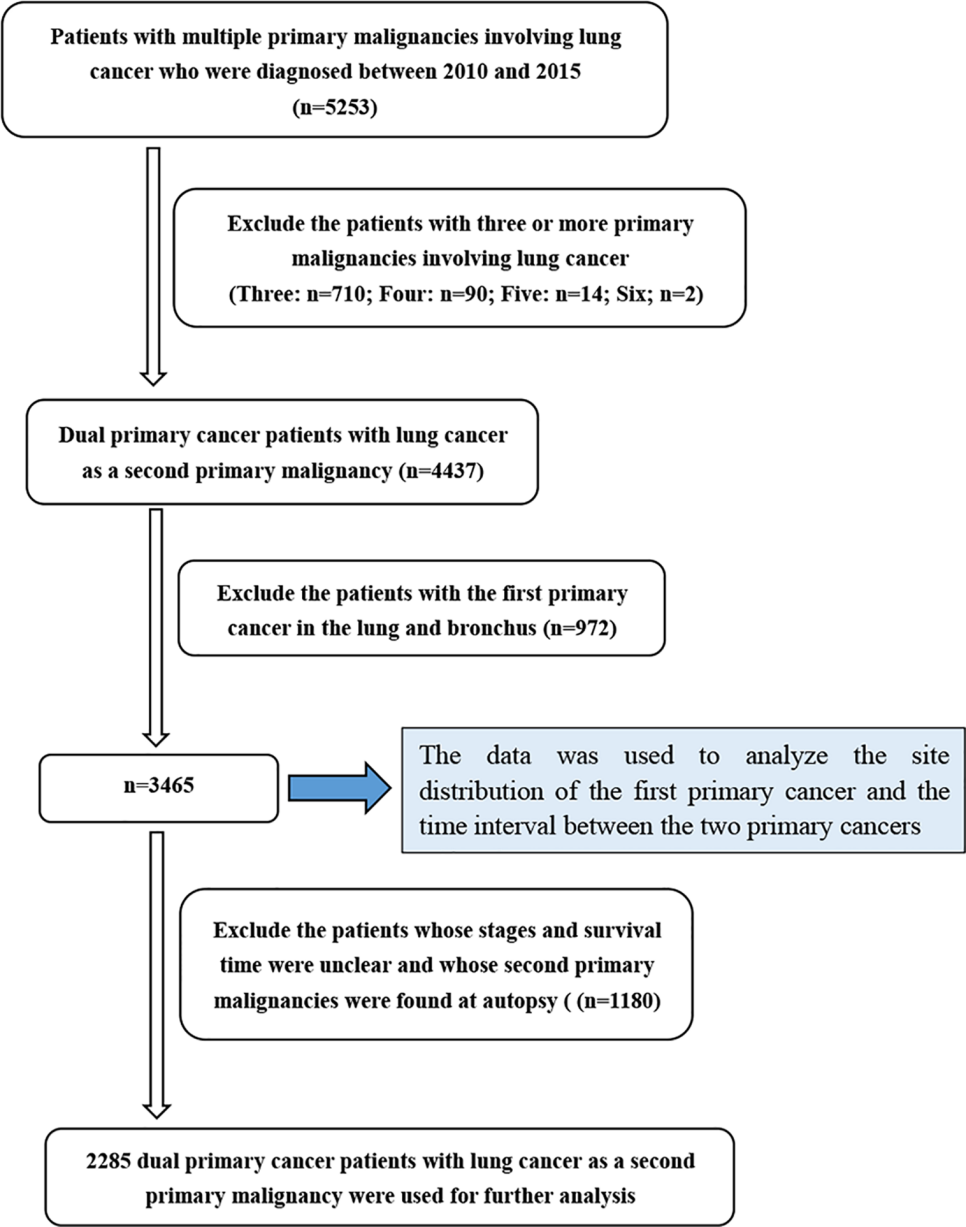
Flow chart detailing the selection of the patients in this study.

The clinical information of LCSPM patients was extracted from the SEER database between 2010 and 2015. We accessed the database using SEER*Stat 8.3.5 software (http://seer.cancer.gov/seerstat/). These data from the SEER database were open to the public for research purposes. This study was also approved by the Institutional Research Committee of Zhongnan Hospital of Wuhan University. We mainly studied the dual primary cancer patients with LCSPM, so cases with three or more primary malignancies were excluded from this study. Given there were still no uniform diagnostic criteria for multiple primary lung cancer (MPLC) and it was difficult to determine whether the second tumor lesion was primary or metastatic, this study also excluded patients with lung cancer as the first primary malignant tumor. The detailed patient selection process is summarized in **Figure 1**. The collected variables included age at diagnosis, sex, “race record,” “ICO-O-3 Hist/behav, malignant,” “month since index” (the time interval between two primary cancers), “Derived AJCC Stage (7^th^ ed),” “COD to site recode,” “Survival months,” “Vital status record (study cutoff used),” “Rx Sumn-Surg Prim Site(1998+),” and “years of diagnosis.”

### Statistical Analysis

In this study, overall survival (OS) was calculated from the diagnosis date of the second primary malignancy (lung cancer) to the date of the last follow-up or death in the SEER database. The OS of all variables were calculated using the Kaplan-Meier method. Survival curves were compared with the log-rank test. Simple random sampling was performed in version 3.6.0 of R software, and patients were randomly divided into a training cohort and a validation cohort at a ratio of 7 to 3. In the training cohort, the Cox proportional hazards model was utilized to estimate OS hazard ratio (HR) for prognostic factors, including age, sex, race, histology, location of FPC, the time interval between two primary cancers, AJCC stage, year of diagnosis, and surgery. All variables were first subjected to univariate Cox analysis, and then variables with *p* < 0.05 were subjected to multivariate Cox analysis. Based on these independent prognostic factors, Kaplan-Meier survival analysis was further performed, and a prognostic nomogram was also constructed to predict 1-, 3-, and 5-year OS rates. The nomogram was developed with the “rms” package in R. In order to evaluate the predictive accuracy of the nomogram, the concordance index (C-index) was calculated by the bootstrap method with 100 resamples, and calibration curves were also drawn simultaneously. Statistics of C-index are generally between 0.5 and 1.0, and the higher the C-index, the higher the predictive value (7). Additionally, decision curve analysis (DCA) widely recommended by many researchers (8, 9), was also used to evaluate the clinical utility of the nomogram in this study.

## Results

### Clinical and Pathological Characteristics

In total, 5253 patients with MPC involving lung cancer were extracted from the SEER database, and 3465 (66.0%) dual primary cancer patients with LCSPM were used to analyze FPC site distribution and the time interval between the two primary cancers. Furthermore, of the 3465 patients, 2285 had complete information and were randomly divided into a training cohort (*n*=1601) and a validation cohort (*n*=684) according to 7:3. Their clinicopathological characteristics are presented in **Table 1**. As can be seen from the table, in the training and validation cohorts, the majority of patients were aged over 65, male, and white. Among these patients, adenocarcinoma had the highest frequency, followed by squamous cell carcinoma (in the entire cohort, 37% of patients presented with adenocarcinoma, 22.3% with squamous cell carcinomas, and 9.3% with small cell cancer). Additionally, patients with stage I lung cancer had a significant proportion in the training and validation cohorts, accounting for 37.6% and 35.2%, respectively. However, in the training and validation cohorts, only 34.6% and 32.4% of patients received lung cancer-directed surgery, respectively. Nonsurgical patients numbered significantly more than surgical patients, accounting for more than 50% in the two cohorts. Additionally, more and more cancer patients were diagnosed with primary lung cancer as the years of diagnosis increased. The incidence rate increased from 3.5% in 2010 to 27.4% in 2015. Given this, we explored the clinicopathological characteristics of patients from 2010 to 2015 (**Table 2**). In every single year, the proportion of men was more than that of women, and adenocarcinoma was still the most common histological type, followed by squamous cell carcinoma. In addition, the proportion of patients with stage I was higher than that of patients with other stages (stage II, III, and IV), and the number of nonsurgical patients was also more than that of surgical patients, and the proportion of nonsurgical patients appeared to be increasing year by year.

**Table 1:** Demographic and clinicopathological characteristics of the training and validation cohorts.

Variables	Entire cohort (n=2285) (N, %)	Training cohort (n=1601) (N, %)	Validation cohort (n=684) (N, %)
**Age (years)**			
<65	622 (27.2)	436 (27.2)	186 (27.1)
>= 65	1663 (72.8)	1165 (72.8)	498 (72.9)
**Sex**			
Female	877 (38.4)	616 (38.4)	261 (38.1)
Male	1408 (61.6)	985 (61.6)	423 (61.9)
**Race**			
White	1862 (81.5)	1303 (81.4)	559 (81.7)
Black	252 (11.0)	175 (10.9)	77 (11.2)
Other	171 (7.5)	123 (7.7)	48 (7.1)
**Histology of lung cancer**			
Adenocarcinoma	863 (37.8)	618 (38.6)	245 (35.8)
Squamous cell carcinomas	510 (22.3)	366 (22.9)	144 (21.1)
Small cell cancer	213 (9.3)	142 (8.8)	71 (10.4)
Others	699 (30.6)	475 (29.7)	224 (32.7)
**Location of FPC**			
Prostate	486 (21.3)	331 (20.6)	155 (22.6)
Female Breast	308 (13.5)	210 (13.1)	98 (14.4)
Urinary Bladder	238 (10.4)	174 (10.9)	64 (9.3)
Others	1253 (54.8)	886 (55.4)	367 (53.7)
**Stage of lung cancer**			
Stage I	843 (36.9)	602 (37.6)	241 (35.2)
Stage II	217 (9.5)	159 (9.9)	58 (8.5)
Stage III	414 (18.1)	298 (18.7)	116 (16.9)
Stage IV	811 (35.5)	542 (33.8)	269 (39.4)
**Surgery**			
No	1511 (66.1)	1048 (65.4)	463 (67.6)
Yes	774 (33.9)	553 (34.6)	221 (32.4)
**Interval (months)**			
<24	1391 (60.9)	986 (61.5)	405 (59.2)
24 - 47	695 (30.4)	479 (30.0)	216 (31.5)
48 - 72	199 8.7)	136 (8.5)	63 (9.3)
**Year of diagnosis**			
2010	81 (3.5)	59 (3.6)	22 (3.3)
2011	228 (10.0)	166 (10.4)	64 (9.3)
2012	344 (15.1)	239 (14.9)	105 (15.4)
2013	436 (19.1)	301 (18.8)	135 (19.7)
2014	569 (24.9)	394 (24.7)	175 (25.6)
2015	625 (27.4)	442 (27.6)	183 (26.7)

**Table 2:** Clinicopathological characteristics of dual primary cancers patients with LCSPM between 2010 and 2015.

Variables	2010 (n = 81)	2011 (n = 230)	2012 (n = 344)	2013 (n = 436)	2014 (n = 569)	2015 (n = 625)
**Age (years)**	68.72 (11.44)	70.50 (10.03)	69.11 (10.60)	70.18 (9.72)	70.41 (9.60)	70.53 (9.60)
**Sex**						
Female	37 (45.68)	90 (39.13)	127 (36.92)	148 (33.94)	212 (37.26)	263 (42.08)
Male	44 (54.32)	140 (60.87)	217 (63.08)	288 (66.06)	357 (62.74)	362 (57.92)
**Histology of lung cancer**						
Adenocarcinoma	28 (34.57)	89 (38.70)	128 (37.21)	176 (40.37)	208 (36.56)	234 (37.44)
Squamous cell carcinomas	16 (19.75)	53 (23.04)	77 (22.38)	93 (21.33)	125 (21.97)	146 (23.36)
Small cell cancer	8 (9.88)	15 (6.52)	37 (10.76)	36 (8.26)	52 (9.14)	65 (10.40)
Others	29 (35.80)	73 (31.74)	102 (29.61)	131 (30.05)	184 (32.34)	180 (28.80)
**Location of FPC**						
Prostate	17 (20.99)	46 (20.0)	78 (22.67)	92 (21.10)	125 (21.97)	128 (20.48)
Female Breast	17 (20.99)	29 (12.61)	39 (11.34)	55 (12.61)	72 (12.65)	96 (15.36)
Urinary Bladder	7 (8.64)	28 (12.17)	34 (9.88)	47 (10.78)	67 (11.78)	55 (8.80)
Others	40 (49.38)	127 (55.22)	193 (56.10)	242 (55.50)	305 (53.60)	346 (55.36)
**Interval (months)**	4.11 (2.44)	8.32 (4.99)	14.93 (9.11)	19.42 (12.34)	24.21 (15.63)	30.41 (19.84)
**Stage of lung cancer**						
Stage I	36 (44.44)	89 (38.70)	126 (36.63)	150 (34.40)	204 (35.85)	238 (38.08)
Stage II	8 (9.88)	25 (10.87)	33 (9.59)	44 (10.09)	41 (7.21)	66 (10.56)
Stage III	11 (13.58)	45 (19.57)	77 (22.38)	71 (16.28)	107 (18.80)	103 (16.48)
Stage IV	26 (32.10)	71 (30.87)	108 (31.40)	171 (39.22)	217 (38.14)	218 (34.88)
**Surgery for lung cancer**						
No	47 (58.02)	141 (61.3)	213 (61.92)	283 (64.91)	395 (69.42)	432 (69.12)
Yes	34 (41.98)	89 (38.7)	131 (38.08)	153 (35.09)	174 (30.58)	193 (30.88)

Continuous variables (age and interval) are presented as mean and standard deviation, and categorical variables are presented as numbers and percentages.

### The Site Distribution of FPC and the Time Interval Between Two Primary Cancers

**Figure 2:**
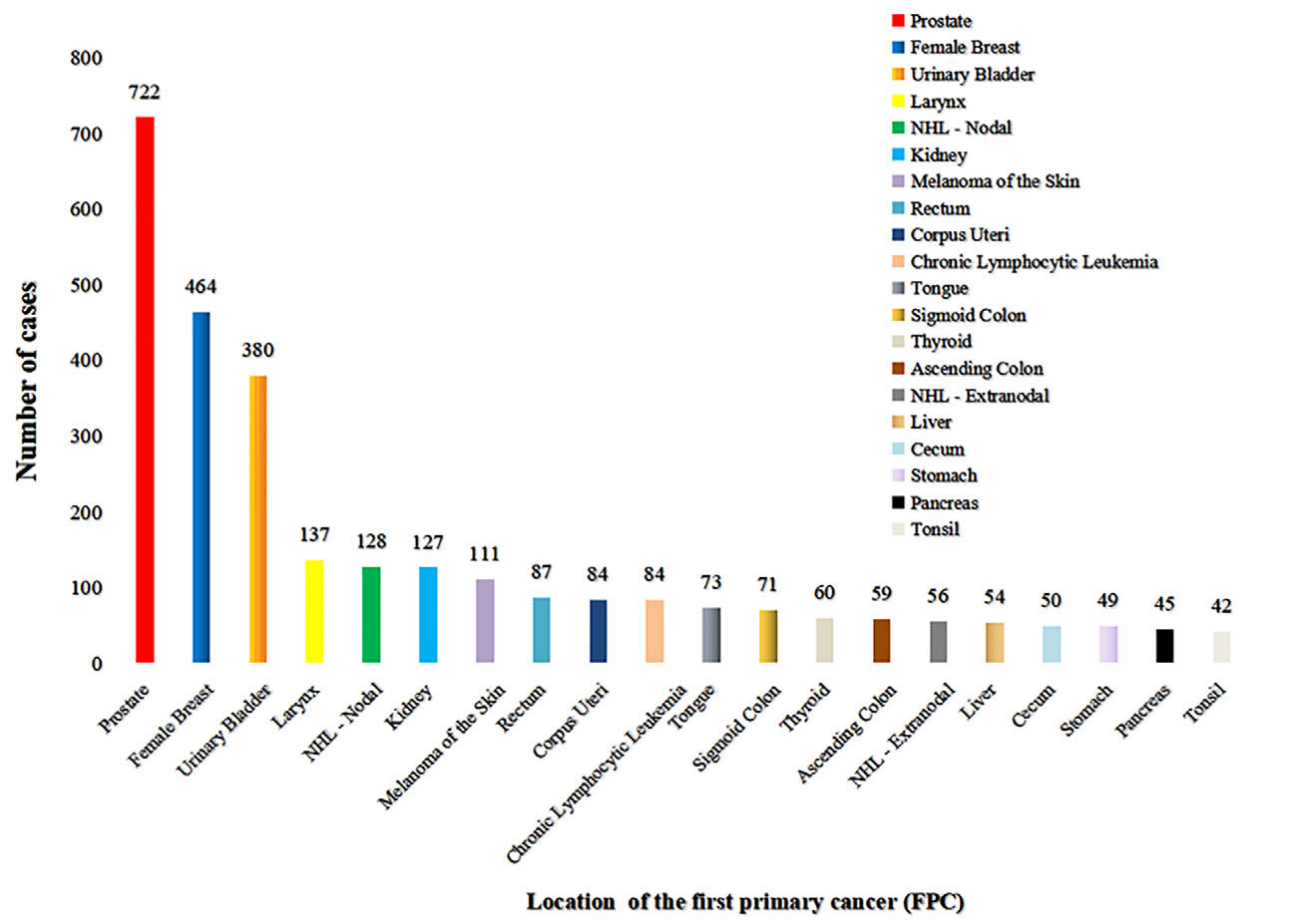
The site distribution of FPC. There were 76 sites of FPC, and the most common site was the prostate (722), followed by female breast (464), and urinary bladder (380) (excluding patients with the first primary cancer in the lung and bronchi). Only the location distribution of more than 40 cases was shown here.

Among the 5253 LCSPM patients, 4437 were dual primary cancers, and 710 were triple primary cancers, 90 were four primary cancers, 14 were five primary cancers, and 2 were six primary cancers (**Figure 1**). There were 76 sites of the FPC, and the most common site was prostate (20.8%), followed by female breast (13.4%) and urinary bladder (11.0%) (**Figure 2** and **Table 3**), for which median interval time was, respectively, 26, 52, and 24 months. Compared with the longest interval of 52 months for female breast cancer patients, patients with pancreatic cancer had the shortest median interval (3.5 months) for developing a primary malignant tumor in the lung (**Table 3**). Additionally, for the entire study population, the median time interval was 21 months (range: 3.5–52 months) as shown in **Table 3**. The time interval of most patients was less than 24 months in the training cohort (61.5%) and validation cohort (59.2%). The proportion of these patients with interval time over 48 months was less than 10% in the two cohorts (**Table 1**).

**Table 3:** Location of the first primary cancer (FPC) and median interval between two primary cancers.

Location of FPC	N (%)	Median interval (months)
Total	3465 (100)	21
Prostate	722 (20.8)	26
Female Breast	464 (13.4)	52
Urinary Bladder	380 (11.0)	24
Larynx	137 (3.95)	9.5
NHL - Nodal	128 (3.69)	17.5
Kidney	127 (3.67)	11
Melanoma of the Skin	111 (3.20)	40.5
Rectum	87 (2.51)	23
Corpus Uteri	84 (2.42)	11.5
Chronic Lymphocytic Leukemia	84 (2.42)	37.5
Tongue	73 (2.11)	18.5
Sigmoid Colon	71 (2.05)	24.5
Thyroid	60 (1.73)	29
Ascending Colon	59 (1.70)	31.5
NHL - Extranodal	56 (1.62)	18.5
Liver	54 (1.56)	30.5
Cecum	50 (1.44)	16
Stomach	49 (1.41)	15
Pancreas	45 (1.30)	3.5
Others	625 (18.1)	8.5

### Prognosis Factors for Overall Survival

**Figure 3:**
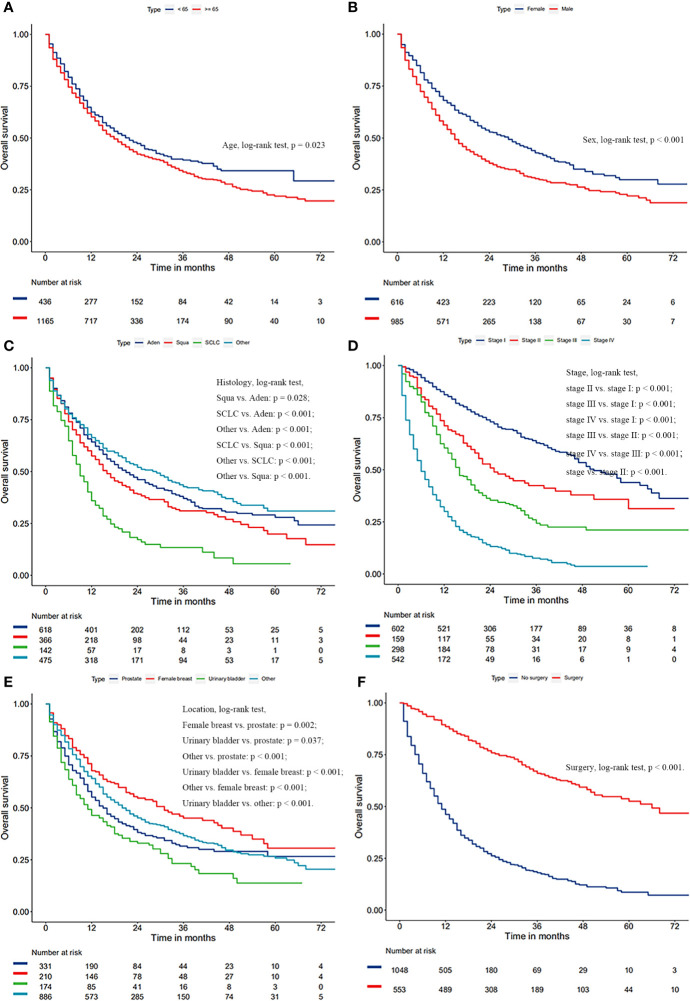
Kaplan-Meier survival curves of overall survival based on age **(A)**, gender **(B)**, histology of lung cancer **(C)**, AJCC stage of lung cancer **(D)**, surgery **(E)**, and location of FPC **(F)**.

After a univariate Cox analysis of 1601 patients in the training cohort, the results showed that age, gender, histology, AJCC stage, FPC location, and surgery were all related to the survival prognosis of these patients (Log-rank test, all *p* < 0.05; **Table 4**). The same finding was also observed in the multivariate Cox analysis. The abovementioned factors were all regarded as independent prognostic factors on which the Kaplan-Meier survival analysis was also further performed as shown in **Figure 3**. It can be seen from **Table 4** and **Figure 3** that the prognosis of patients over 65 years old was worse than that of patients under the age of 65 (HR = 1.18, *p* = 0.024) and 3-year OS rates were 33.6% and 39.3%, respectively (log-rank test, *p* = 0.023). Men were associated with a worse 3-year OS compared to women (30.4% vs. 42.8%, *p* < 0.001). The later the stage of lung cancer, the worse the prognosis (log-rank test, *p* < 0.001). Lung cancer-directed surgery could significantly improve long-term survival (HR = 0.22, p < 0.001). The prognosis of patients with urinary bladder cancer as FPC was the worst in the Kaplan-Meier survival analysis, univariate, and multivariate Cox analysis (log-rank test, all *p* < 0.05). The prognosis of patients with squamous cell carcinoma was between small cell lung cancer (SCLC) and adenocarcinoma, and 3-year OS rates were 30.7%, 11.8%, and 37.0%, respectively (log-rank test, all *p* < 0.05).

**Table 4:** Univariate and multivariate Cox analysis for these patients in the training cohort.

Variables	Univariate Cox analysis	P value	Multivariate Cox analysis	P value
	HR (95% CI)		HR (95% CI)	
**Age** (years)				
<65	Reference		Reference	
>= 65	1.18 (1.02-1.36)	0.024	1.25 (1.08-1.45)	0.003
**Sex**				
Female	Reference		Reference	
Male	1.45 (1.27-1.65)	<0.001	1.28 (1.08-1.51)	0.004
**Race**				
White	Reference		—	
Black	0.98 (0.80-1.20)	0.865	—	
Other	1.07 (0.85-1.35)	0.558	—	
**Histology of lung cancer**				
Adenocarcinoma	Reference		Reference	
Squamous cell carcinomas	1.21 (1.02-1.42)	0.024	1.21 (1.03-1.43)	0.022
Small cell cancer	2.13 (1.73-2.62)	<0.001	1.34 (1.08-1.65)	0.007
Others	0.87 (0.74-1.02)	0.089	1.13 (0.96-1.32)	0.147
**Location of FPC**				
Prostate	Reference		Reference	
Female Breast	0.67 (0.53-0.84)	<0.001	1.21 (0.91-1.61)	0.199
Urinary Bladder	1.29 (1.03-1.61)	0.024	1.53 (1.23-1.92)	<0.001
Others	0.85 (0.72-0.99)	0.046	1.35 (1.13-1.61)	<0.001
**Stage of lung cancer**				
Stage I	Reference		Reference	
Stage II	1.74 (1.35-2.23)	<0.001	1.80 (1.44-2.32)	<0.001
Stage III	2.70 (2.23-3.29)	<0.001	1.80 (1.46-2.21)	<0.001
Stage IV	6.36 (5.39-7.51)	<0.001	3.90 (3.24-4.70)	<0.001
**Surgery**				
No	Reference		Reference	
Yes	0.22 (0.18-0.25)	<0.001	0.36 (0.30-0.44)	<0.001
**Interval (months)**				
<24	Reference		—	
24 - 47	1.08 (0.94-1.24)	0.277	—	
48 - 72	0.86 (0.65-1.13)	0.276	—	
**Year of diagnosis** (year)				
2010	Reference		—	
2011	1.03 (0.74-1.45)	0.852	—	
2012	1.06 (0.76-1.47)	0.741	—	
2013	1.14 (0.83-1.58)	0.424	—	
2014	1.06 (0.77-1.47)	0.702	—	
2015	0.88 (0.63-1.22)	0.441	—	

**Figure 4:**
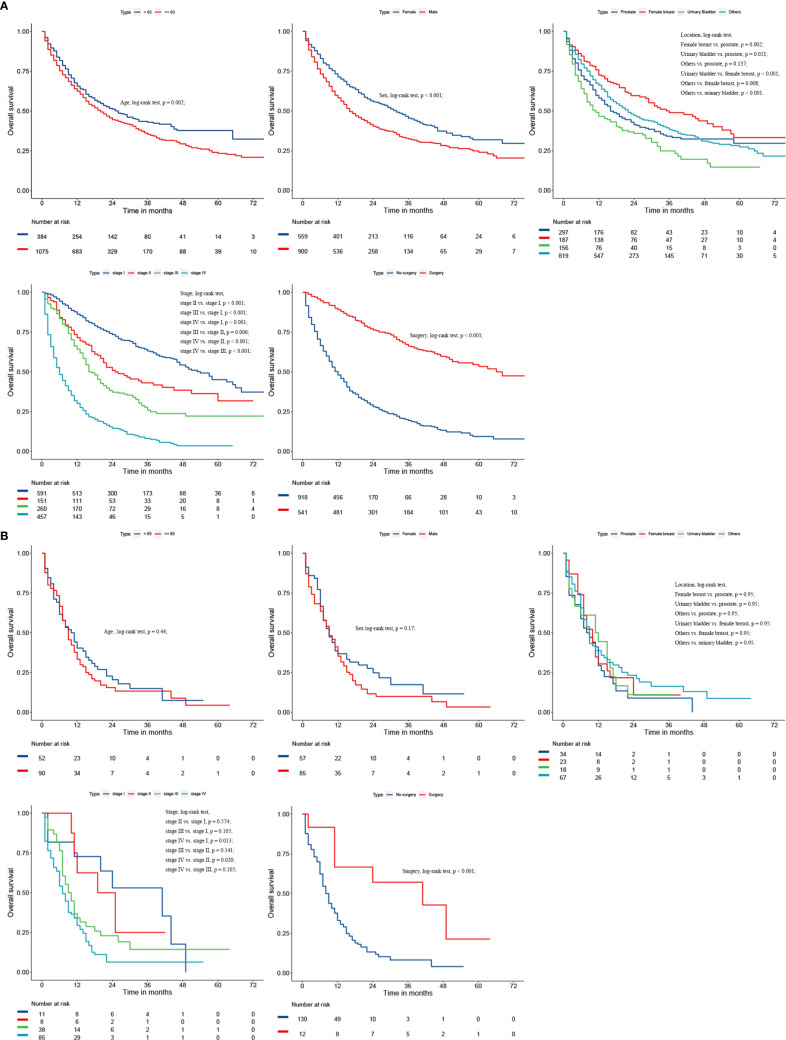
Kaplan-Meier survival curves of overall survival for NSCLC **(A)** and SCLC **(B)**.

Considering the great difference in biological behavior and prognosis between NSCLC and SCLC, we separately analyzed the survival of these patients. Age, gender, AJCC stage, FPC location, and surgery were all regarded as related to the survival prognosis of NSCLC patients (log-rank test, all *p* < 0.05; **Figure 4** and **Table S1**). However, for patients with SCLC as a second primary malignant tumor, age, gender, and FPC location did not affect the prognosis, and surgery alone was considered to be an independent prognostic factor for patients (**Figure 4** and **Table S1**). In addition, in the univariate Cox analysis, we found that the time interval between two primary cancers was not related to the long-term survival of NSCLC and SCLC patients (all *p* > 0.05). There was also no correlation between the prognosis and the year of diagnosis (all *p* > 0.05).

### Development and Validation of the Prognosis Nomogram

**Figure 5:**
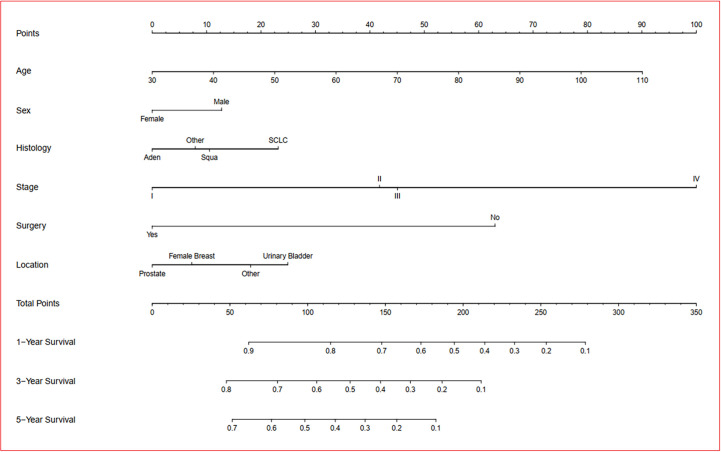
Prognostic nomogram of overall survival in dual primary cancer patients with LCSPM. Nomogram to predict 1-, 3-, and 5-year OS rates of the patients. The factors of age, sex, histology, stage, location of FPC, and surgery were included in the model. Aden: adenocarcinoma; Squa: squamous cell carcinomas; SCLC: small cell lung cancer.

**Figure 6:**
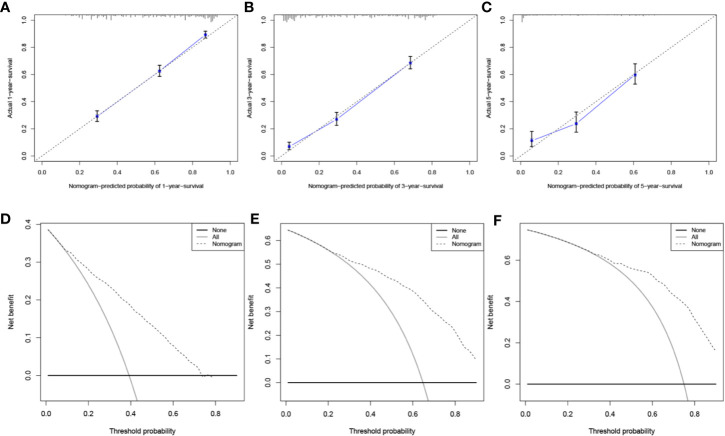
Evaluation of the prognostic nomogram. Calibration curves for 1-year **(A)**, 3-year **(B)**, and 5-year **(C)** OS in the training cohort. DCA curves for 1-year **(D)**, 3-year **(E)**, and 5-year **(F)** OS in the training cohort.

A prognosis nomogram was developed to predict 1-, 3-, and 5-year OS rates on the basis of 1601 patients in the training cohort. The established nomogram included all statistically significant prognostic factors in the Cox proportional hazard model, involving age, gender, histology, AJCC stage, FPC location, and surgery (**Figure 5**). According to the different classifications of each characteristic, points were projected upward to get the score of each item. The total points were calculated by adding all the points, and then the survival rate of patients were calculated by projecting the total points downward. The higher the score was, the worse the survival prognosis was. This nomogram can be used to predict the survival rate of different patients according to their own conditions, thereby improving the efficiency and accuracy of prediction. In this study, the established nomogram was verified by the bootstrap method with 100 resamples in the training (*n*=1601) and validation (*n*=684) cohorts. The C-index of internal validation was 0.760 (95% CI: 0.744–0.776), and that of external validation was 0.759 (95% CI: 0.737–0.781). The corresponding calibration curves of 1-, 3-, and 5-year OS rates in training and validation cohorts are also shown in **Figures 6** and **S1**, from which we can see that all calibration curves are close to the ideal 45° dotted line. This indicates that the predicted value of the model had good consistency with the actual observed value. In addition, all DCA curves in training and validation cohorts also indicated the model had relatively ideal clinical utility (**Figures 6** and **S1**).

## Discussion

In recent years, with the continuous advancement of medical technology and the improvement of patient compliance, many cancer patients have been diagnosed with new primary malignant tumors in their lungs. In the past, a large number of studies have focused on single primary lung cancer or multiple primary lung cancer (MPLC), but there are few studies on lung cancer patients with other primary malignancies. To date, little is known about the regularity of the time interval between two primary malignancies and the prognosis of dual primary cancer patients with LCSPM. Thus, this study retrospectively analyzed the clinical characteristics of 3465 dual primary cancer patients with LCSPM extracted from the SEER database between 2010 and 2015, intending to improve the understanding of these diseases and provide a certain reference for future clinical work.

During the follow-up of cancer patients, clinicians tend to focus more on the organ where the primary tumor is located and other organs where the tumor is more likely to metastasize, which will inadvertently ignore the risk of developing a primary malignancy in other organs. Lung cancer, a malignant tumor with a high incidence rate and mortality rate, poses a serious threat to public health. Thus, it is of great clinical significance to clarify the common sites of FPC in LCSPM patients to improve the effectiveness of follow-up and vigilance of cancer patients. Through analysis of 185 patients with MPC involving lung cancer from Guangdong Lung Cancer Research Institute from 2005 to 2013, Li et al. found that colorectal cancer, esophageal cancer, and thyroid cancer were the tumors that most frequently accompanied lung cancer (10). Liu et al. also reported that the most common tumors associated with lung cancer were upper aerodigestive tract cancer, colorectal cancer, and cervical cancer (1). In this study, we found that, in 3465 dual primary cancer patients with LCSPM, the most common organ of FPC was prostate, followed by female breast, and urinary bladder, accounting for 20.8%, 13.4%, and 11.0%, respectively. Obviously, the findings of these studies were significantly different. We believe that, in addition to the different sample size, the reasons for this phenomenon might also be related to geographical environment (China/American), ethnic differences, and research design (different from them, the cases with FPC in the lung and bronchus were excluded in our study). Despite the differences, all the findings suggest that cancer patients were still at risk of developing new primary malignant tumors in their lungs. Thus, cancer patients, as well as clinicians, should pay close attention to the changes of the lung or other organs and be alert to the occurrence of lung cancer or other malignant tumors during follow-up. Of course, we should also note that periodic follow-up to find a new primary tumor in the lung is a kind of cancer screening for high-risk populations. These patients usually have a long history of smoking, exposure to chemicals, family history of lung cancer, etc.

Definitely, understanding the time interval between two primary cancers can assist clinicians to develop better follow-up strategies for cancer patients. Li and his colleagues found that the median interval between two primary cancers in MPC patients was 41.2 months (10). Liu et al. also observed that, when lung cancer was the second primary cancer, the interval time between the two primary malignancies was 46 months (1). The findings were longer than that of our study (the median interval was 21 months in our study), which may be related to the inclusion criteria and sample size of the study. Because there was no recognized diagnostic criteria for MPLC, our study excluded the cases with lung cancer as FPC and included 3465 dual primary cancer patients with LCSPM from the SEER database with significantly more cases than other studies (there were only 185 cases in Li’s study and 142 cases in Liu’s study). To the best of our knowledge, this is one of the largest studies on this topic. In daily clinical practice, how long and how often to follow up after the diagnosis of FPC is a matter in hand. Our study found that the median interval between the FPCs (prostate cancer, female breast cancer, and urinary bladder cancer) and lung cancer (the second primary cancer) were 26 months, 52 months, and 24 months, respectively. Additionally, for the entire study cohort, the median interval between the FPC and second primary cancer (lung cancer) was 21 months, the shortest interval was 2 months, and the longest was 81 months. This indicates that patients with cancer are still at the risk of developing another new primary malignancy in the lungs. Close, lifelong follow-up was recommended for all cancer patients not only to detect recurrence or metastasis, but also to detect early-stage primary tumors in the lungs or other organs.

In this study, we observed that age, sex, histology, stage, and surgery were all closely related to the prognosis of these patients. Advanced age (> 65 years old) and being male were independent risk factors for patients. Compared with nonsurgical treatment, lung cancer-directed surgery could significantly improve OS of these patients, with 3-year OS rates of 18.0% and 66.0%, respectively. SCLC had the worst prognosis. The later the stage of lung cancer, the worse the prognosis. This was also in line with the findings of other studies (11, 12). Massard et al. (11) reported that the survival of LCSPM patients was associated with the stage of lung cancer. Kim et al. (12) also found advanced lung cancer stage was a poor prognostic factor for patients with MPC involving lung cancer. In addition, some retrospective research has demonstrated that patients with MPC involving lung cancer tended to have the better long-term survival than ordinary lung cancer population (1, 4, 13). However, so far there are few studies on whether the prognosis of LCSPM is related to another primary malignancy. This study found that the 3-year OS of LCSPM patients with urinary bladder cancer as FPC was significantly lower than that of patients with other primary malignancies as FPC. It should be noted that lung cancer here referred only to NSCLC, and the prognosis of dual primary cancer patients with SCLC as a second primary malignancy had no relation to the FPC. Kim et al. (12) observed that cancer patients with another primary malignancy in the head and neck tended to have a worse prognosis than these patients with another primary malignancy elsewhere. Unfortunately, due to so few cases (less than 1.3%) with FPC in the head and neck, our study did not separately compare the prognosis of these patients with those of other patients, which may result in different results.

Additionally, our study found that, since 2010, more and more cancer patients were diagnosed with another new primary tumor in their lungs. This trend was mainly related to the following points. First, the age of the population was prolonged. Second, more and more chemicals were coming into contact. The third was the influence of bad habits, such as cigarettes. The fourth were the advances in imaging technology and the increasing pace of life. Finally, an important factor was the increasing awareness of early lung cancer screening. Several studies (14, 15) have demonstrated that cancer patients, compared to the general population, had a higher risk to develop new primary tumors. Therefore, we believe that, even if the primary tumor has undergone radical surgery, the cancer patient still needs long-term close follow-up. In addition to paying attention to changes in the organ where the primary tumor is located, changes in other organs should not be ignored.

Good prognosis evaluation is of great significance for the treatment and follow-up of cancer patients. Clinically, due to the lack of a relatively perfect scoring system, clinicians often make empirical judgments based on the patients’ age, AJCC stage and pathological results. As an emerging tool widely used in some clinical research (5, 6, 16), a nomogram can integrate the influence of various prognostic factors in the clinic and present the results visually. Compared with traditional methods, it can make predictions more quickly and accurately, and its predictive value has been considered superior to other evaluation systems (17, 18). Thus, a prognosis nomogram was also applied in this study. From the established nomogram, we could intuitively see the influence of each independent prognostic factor on score points. Considering the good prediction performance and clinical utility of this nomogram were fully proven in both internal and external validation sets, this clinical nomogram is expected to be routinely applied to the survival prediction of such patients in the future.

Our study has the following advantages. First, we used the large sample size of the SEER database to determine the common sites of FPC and the median interval between the two primary malignancies in dual primary cancer patients with LCSPM, which was of great significance in improving the effectiveness of follow-up in cancer patients. Second, our study was the first attempt to use a nomogram to predict the survival of dual primary cancer patients with LCSPM, which included 2285 patients from the SEER database, and its data accuracy was up to 95% (19). Third, our preliminary findings can help clinicians understand this disease better and serve as a basis for future, larger multicenter studies.

Admittedly, our study also has some shortcomings. First, the limitations of the SEER database widely discussed in previous studies (20, 21). Second, research on MPC involving lung cancer is still lacking, and thus, the understanding of this special population remains limited. Although this is a multicenter study with a large sample size, this SEER-based study can still not provide important survey information on the risk of multiple primary cancer due to the limitations of the SEER database, including smoking status, genetic conditions (such as gene mutation), family history, exposure history (chemicals), organ transplantation, or chronic immunosuppression to name a few. In the end, this study, as a retrospective analysis, inevitably leads to selective bias. Taking into account the deficiencies of retrospective research, prospective analysis is recommended to proceed further.

## Conclusion

In summary, dual primary cancer patients with LCSPM have approximately 59.3% of 1-year OS, 34.7% of 3-year OS, and 25.2% of 5-year OS, respectively. Systematic and periodic follow-up is recommended for all cancer patients, and other organs should not be ignored in the follow-up of cancer patients. Early detection for surgical treatment will significantly improve the prognosis of these patients.

## Data Availability Statement

Publicly available datasets were analyzed in this study. This data can be found here: http://seer.cancer.gov/data/.

## Ethics Statement

The present study was approved by the Institutional Research Committee of Zhongnan Hospital of Wuhan University. Written informed consent for participation was not required for this study in accordance with the national legislation and the institutional requirements.

## Author Contributions 

CS, WH, and SL: designed the study. CS, SL, and WH: reviewed relevant literature and drafted the manuscript. CS, QW, and YW conducted all statistical analyses. All authors contributed to the article and approved the submitted version.

## Funding

This work was supported by Key Projects of Hubei Provincial Health and Family Planning Commission (WJ2017Z006) and Zhongnan Hospital of Wuhan University Science Technology and Innovation Cultivating Fund (cxpy2017041) and the 351 Talent Project of Wuhan University (Luojia Yong Scholars: SL).

## Conflict of Interest

The authors declare that the research was conducted in the absence of any commercial or financial relationships that could be construed as a potential conflict of interest.

## Supplementary Material

Supplementary Figure 1 Evaluation of the prognostic nomogram. Calibration curves for 1-year **(A)**, 3-year **(B)**, and 5-year **(C)** OS in the validation cohort. DCA curves for 1-year **(D)**, 3-year **(E)**, and 5-year **(F)** OS in the validation cohort.

Supplementary Table 1 Univariate and multivariate Cox analysis for NSCLC and SCLC patients in the training cohort.

## Abbreviations

MPC, multiple primary cancers; MPLC, multiple primary lung cancers; LCSPM, lung cancer as a second primary malignancy; FPC, first primary cancer; SCLC, small cell lung cancer; NSCLC, non-small cell lung cancer; OS, overall survival; HR, hazard ratio; SEER, Surveillance, Epidemiology, and End Results.
