# Supplementary figures and images for: Dual Primary Cancer Patients With Lung Cancer as a Second Primary Malignancy: A Population-Based Study
Source: Front Oncol. 2020 Oct 26;10:515606. doi: 10.3389/fonc.2020.515606 (PMC7649344; doi:10.3389/fonc.2020.515606)

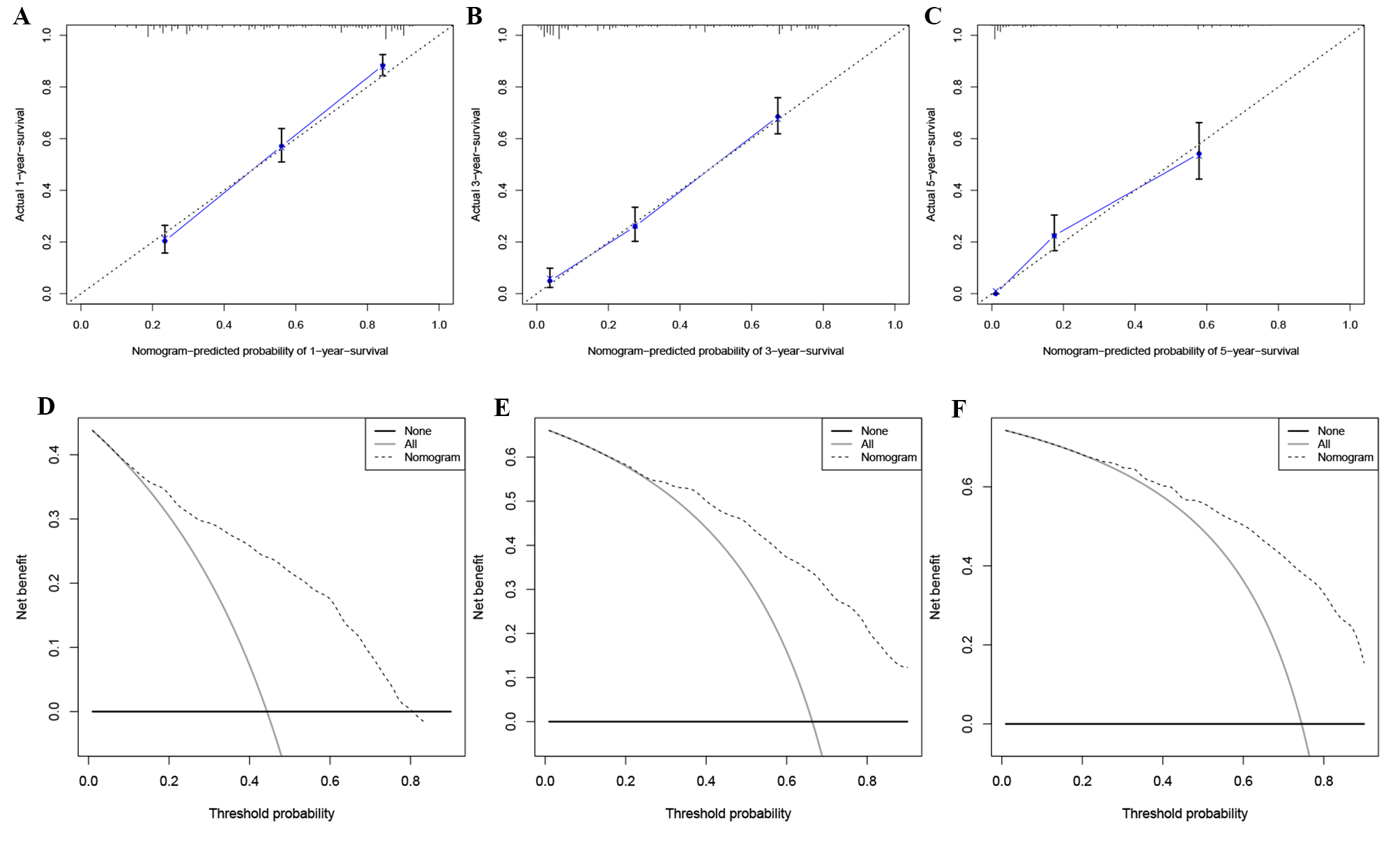

Supplement: Supplementary Figure 1 — Evaluation of the prognostic nomogram. Calibration curves for 1-year (A), 3-year (B), and 5-year (C) OS in the validation cohort. DCA curves for 1-year (D), 3-year (E), and 5-year (F) OS in the validation cohort. [file Image_1.tif]
