# Supplementary material for: Dual Primary Cancer Patients With Lung Cancer as a Second Primary Malignancy: A Population-Based Study
Source: Front Oncol. 2020 Oct 26;10:515606. doi: 10.3389/fonc.2020.515606 (PMC7649344; doi:10.3389/fonc.2020.515606)
Supplement: Supplementary Table 1 — Univariate and multivariate Cox analysis for NSCLC and SCLC patients in the training cohort. [file Table_1.doc]

**Table S1:** Univariate and multivariate Cox analysis for NSCLC and SCLC patients in the training cohort.

| **Variables** | **NSCLC** | | | | **SCLC** | | | |
| --- | --- | --- | --- | --- | --- | --- | --- | --- |
|  | Univariate Cox analysis | | Multivariate Cox analysis | | Univariate Cox analysis | | Multivariate Cox analysis | |
|  | HR (95% CI) | P value | HR (95% CI) | P value | HR (95% CI) | P value | HR (95% CI) | P value |
| **Age** (years) |  |  |  |  |  |  |  |  |
| < 65 | Reference |  | Reference |  | Reference |  | --- |  |
| >= 65 | 1.24 (1.06 -1.45) | 0.008 | 1.25 (1.07-1.47) | 0.005 | 1.16 (0.80-1.69) | 0.431 | --- |  |
| **Sex** |  |  |  |  |  |  |  |  |
| Female | Reference |  | Reference |  | Reference |  | --- |  |
| Male | 1.47 (1.28-1.70) | <0.001 | 1.28 (1.07-1.52) | 0.006 | 1.30 (0.89-1.88) | 0.174 | --- |  |
| **Race** |  |  |  |  |  |  |  |  |
| White | Reference |  | --- |  | Reference |  | --- |  |
| Black | 1.02 (0.83 -1.26) | 0.852 | --- |  | 0.75 (0.39-1.45) | 0.396 | --- |  |
| Others | 1.12 (0.88 -1.43) | 0.362 | --- |  | 0.78 (0.36-1.68) | 0.526 | --- |  |
| **Location of FPC** |  |  |  |  |  |  |  |  |
| Prostate | Reference |  | Reference |  | Reference |  | --- |  |
| Female Breast | 0.64 (0.50-0.83) | <0.001 | 1.15 (0.85-1.57) | 0.369 | 0.80 (0.45-1.43) | 0.451 | --- |  |
| Urinary Bladder | 1.35 (1.07-1.71) | 0.013 | 1.66 (1.31-2.11) | <0.001 | 0.83 (0.45-1.52) | 0.542 | --- |  |
| Others | 0.87 (0.74-1.04) | 0.136 | 1.40 (1.16-1.69) | <0.001 | 0.74 (0.47- 1.16) | 0.193 | --- |  |
| **Stage of lung cancer** |  |  |  |  |  |  |  |  |
| Stage I | Reference |  | Reference |  | Reference |  | Reference |  |
| Stage II | 1.75 (1.35-2.26) | <0.001 | 1.93 (1.49-2.50) | <0.001 | 0.98 (0.32-3.01) | 0.970 | 0.72 (0.23 -2.29) | 0.577 |
| Stage III | 2.58 (2.11-3.16) | <0.001 | 1.78 (1.44-2.21) | <0.001 | 2.03 (0.92-4.46) | 0.078 | 1.34 (0.57 - 3.18) | 0.503 |
| Stage IV | 6.41 (5.39-7.61) | <0.001 | 4.01 (3.31-4.85) | <0.001 | 3.14 (1.48-6.63) | 0.003 | 1.98 (0.86 - 4.59) | 0.110 |
| **Surgery** |  |  |  |  |  |  |  |  |
| No | Reference |  | Reference |  | Reference |  | Reference |  |
| Yes | 0.22 (0.19-0.26) | <0.001 | 0.37 (0.30-0.45) | <0.001 | 0.29 (0.13-0.63) | 0.002 | 0.41 (0.17-0.99) | 0.048 |
| **Interval (months)** |  |  |  |  |  |  |  |  |
| < 24 | Reference |  | --- |  | Reference |  | --- |  |
| 24 - 47 | 1.12 (0.96-1.29) | 0.147 | --- |  | 0.83 (0.55-1.26) | 0.393 | --- |  |
| 48 - 72 | 0.81 (0.60-1.10) | 0.178 | --- |  | 1.33 (0.69- 2.58) | 0.391 | --- |  |
| **Year of diagnosis** |  |  |  |  |  |  |  |  |
| 2010 | Reference |  | --- |  | Reference |  | --- |  |
| 2011 | 1.03 (0.72-1.47) | 0.880 | --- |  | 1.39 (0.47-4.06) | 0.552 | --- |  |
| 2012 | 0.99 (0.70-1.41) | 0.973 | --- |  | 2.03 (0.77-5.35) | 0.154 | --- |  |
| 2013 | 1.13 (0.81-1.60) | 0.472 | --- |  | 1.44 (0.53-3.85) | 0.474 | --- |  |
| 2014 | 1.02 (0.72-1.43) | 0.927 | --- |  | 1.90 (0.73-4.94) | 0.190 | --- |  |
| 2015 | 0.84 (0.59-1.19) | 0.327 | --- |  | 1.61 (0.61-4.26) | 0.338 | --- |  |
